# Low-Level Auditory Processing Correlates With Language Abilities: An ERP Study Investigating Sequence Learning and Auditory Processing in School-Aged Children

**DOI:** 10.1162/nol_a_00129

**Published:** 2024-06-03

**Authors:** Anna Kautto, Henry Railo, Elina Mainela-Arnold

**Affiliations:** Department of Psychology and Speech-Language Pathology, University of Turku, Turku, Finland

**Keywords:** auditory ERPs, capacity theories, language development, late talking, procedural deficit hypothesis

## Abstract

Auditory processing and procedural learning deficits have been associated with language learning difficulties. We investigated the relationship of these skills and school-age language abilities in children with and without a history of late talking using auditory event related potentials (ERPs). Late talking (i.e., slow early language development) increases the risk of persistent language difficulties, but its causes remain unknown. Participants in this study were children with varying language abilities (*n* = 60). Half of the participants (*n* = 30) had a history of late talking. We measured procedural learning by manipulating the predictability of sine tone stimuli in a passive auditory ERP paradigm. Auditory processing was tested by examining how the presence of noise (increasing perceptual demands) affected the ERPs. Contrary to our hypotheses on auditory processing and language development, the effect of noise on ERPs did not correlate with school-age language abilities in children with or without a history of late talking. Our paradigm failed to reveal interpretable effects of predictability leaving us unable to assess the effects of procedural learning. However, better language abilities were related to weaker responses in a 75–175 ms time window, and stronger responses in a 150–250 ms time window. We suggest that the weak early responses in children with better language ability reflect efficient processing of low-level auditory information, allowing deeper processing of later, high-level auditory information. We assume that these differences reflect variation in brain maturation between individuals with varying language abilities.

## INTRODUCTION

While most children acquire first language with no difficulties, for some children, the development is slow, or persistently deficient. Theories about underlying neurocognitive mechanisms have been suggested to explain individual differences in language development. In this article, we report findings from an electroencephalogram (EEG) study that was designed to test if auditory processing or procedural learning explain individual differences in language development. Knowledge about the neural basis of language learning is needed to effectively prevent and treat persistent language learning difficulties that are known to affect academic performance and quality of life.

### Language Development and Its Difficulties

First language is typically acquired during the early years without deliberate effort by the child or their caregivers. In addition to sufficient language input, implicit cognitive processes are crucial for language development, and it is assumed that impairments in these processes result in language learning difficulties. Late talkers are children whose language is slower than usual during the first years of life. A common characteristic of late talking (LT) is limited expressive vocabulary at 2 years of age. LT has a relatively good prognosis, and most of these children catch up the delay to their peers by school age (Bishop et al., 2016; Rescorla, 2011). However, for some children with LT, the difficulties persist, and many children that meet the diagnostic criteria for developmental language disorder (DLD) exhibit a history of late talking.

DLD (formerly also referred to as specific language impairment) refers to a condition in which language problems persist past the age of four, and have significant impact on everyday social interactions and educational progress (Bishop et al., 2017). DLD often co-occurs with other neurodevelopmental difficulties, such as attentional and behavioral problems, or motor impairments (Bishop et al., 2016). Although the origins of DLD are unknown, many studies have tested mechanisms that might explain it (Ebert & Kohnert, 2011; Kujala & Leminen, 2017; Lum et al., 2012; Miller et al., 2001; Tallal & Piercy, 1975).

Rather than clear categories of typical and atypical, language abilities are a continuum varying from very weak to very strong, and cut-off points used in defining a disorder are often more or less arbitrary (e.g., Lancaster & Camarata, 2019). Studies on children with LT have also suggested that while most catch up their peers in language development, they may still continue to perform below average on language measures (Rescorla, 2009). In this study, we recruited participants with and without a history of LT, and treated language abilities as a continuum to better represent school-age language outcomes of these children. Nevertheless, it is likely that differences in cognition underlying language difficulties that meet the diagnostic criteria of DLD, are similar to differences contributing to overall variation in these skills. To this point, theories of DLD introduced below also provide useful tools to explain variation in language abilities in children without DLD.

Studies suggest that various mechanisms, such as constraints in working memory (Leonard, 2014; Leonard et al., 2007), temporal processing difficulties (Merzenich et al., 1996; Tallal & Piercy, 1975), general slowness (Kail, 1994), procedural deficit (Ullman & Pierpont, 2005), or auditory processing difficulties (Corriveau et al., 2007) underlie individual differences in language development. Auditory processing accounts on DLD suggest that language learning difficulties arise from restrictions in processing auditory input, such as spoken language. Tallal and Piercy (1975) suggested that children with DLD exhibit difficulties especially in processing rapid auditory stimuli, characteristic for spoken language. However, auditory processing difficulties in DLD are not limited to brief and rapidly successive acoustic cues (Corriveau et al., 2007). Evidence for auditory processing difficulties in DLD have been reported in both behavioral studies (e.g., judging the similarity or order of tone stimuli; see McArthur & Bishop, 2001) and studies measuring brain activity during listening (e.g., mismatch negativity; see Kujala & Leminen, 2017).

According to the procedural deficit hypothesis (PDH; Ullman & Pierpont, 2005), DLD is not explained by auditory processing limitations, but by a domain-general procedural memory deficit instead. The hypothesis posits that atypical development of brain structures supporting procedural memory results not only in limitations in processing motor—as well as cognitive patterns and regularities—but also in rule-based aspects of language, such as phonology and morphosyntax. The procedural deficit is suggested to be domain general in nature. A range of different tasks has been used in exploring procedural learning in DLD. These tasks include auditory statistical learning (e.g., Evans et al., 2009), probabilistic categorization (e.g., Kemény & Lukács, 2010), and artificial grammar learning tasks (e.g., Gillis et al., 2022). The most common task used to test PDH has been the serial response time (SRT) task, a visuomotor task that measures pattern learning. In an SRT task, the participant is following visual stimuli appearing in one of multiple possible locations and is asked to respond with a button press corresponding to the stimulus location as soon as the stimulus appears. Stimulus locations follow a pattern of which the participant is unaware. The task effect is observed in the shortening of response times as the participant implicitly learns the sequence, and response times typically become longer again when the stimuli start to appear in random order. Weak performance in this task has been associated with DLD (see Lum et al., 2014). Thus, most previous studies that report associations of procedural learning and language abilities used behavioral tasks measuring motor learning (e.g., Lum et al., 2014; Sanjeevan et al., 2018). In our study, we aimed to extend the findings on procedural learning and language to a passive, nonmotor task using an event-related potential (ERP) paradigm decribed later in the Introduction.

### ERP Paradigms in Studying Language

EEG provides a valuable tool to study language-related phenomena because of its good temporal resolution. ERP studies have employed mismatch negativity (MMN) to investigate neural auditory discrimination in children with DLD (see Kujala & Leminen, 2017). MMN is an early automatic response occurring approximately 200 ms after stimulus onset to deviations in physical properties of stimuli (Näätänen, 1992), and it reflects relatively early stages of information processing (Escera et al., 2014). As an automatic response, it places low demand on cooperation and thus provides a valuable method to study auditory processing in young children. Auditory mismatch responses are measured using the oddball paradigm. In this paradigm, the stimulus sequences consist of repeating standard stimuli and infrequent deviant stimuli. Deviant stimuli violate the regularity of standard stimuli and elicit mismatch responses in the brain. MMN studies have reported atypical low-level neural discrimination in DLD, such as smaller response amplitudies and longer latencies (for review, see Kujala & Leminen, 2017). Factors known to affect auditory MMN include physical properties of stimuli (e.g., length, intensity, complexity, or pitch), linguistic meaning, MMN design (the difference between standard and deviant and the probability of deviants), interstimulus intervals (ISIs), electrode locations, participant age or maturational level, task (active or passive), and linguistic meaning, among others (Näätänen, 1992). Of these factors, at least MMN for tone pitch (e.g., Holopainen et al., 1998), consonant changes in syllables (Uwer et al., 2002), and the lateralization of MMN (Rinker et al., 2007) have been reported to differ between children with and without DLD. General ERP waveforms have also been reported to differ between children with and without language disorders, ERPs of at least some children with DLD resembling those of younger children with typical language development (Bishop & McArthur, 2005). The observed differences in auditory processing have been suggested to reflect a possible mechanism accounting for language learning difficulties (see Bishop, 2007).

#### MMN and ERPs in studying auditory processing

In this study, we measured MMN to sine tones in silence and noise. Following the auditory processing theories of DLD, we hypothesized that the detrimental effect of noise on MMN responses would be more pronounced in participants with lower language abilities compared to those with higher language abilities. Additionally, we investigated differences in the effect of noise on MMN responses based on participants’ history of LT, with the aim of elucidating possible mechanisms underlying this phenomenon. We examined whether the noise effect of auditory processing (i.e., the extent to which MMN is modified by noise) would be associated differently with language outcomes in children with and without a history of LT. With the noise manipulation, we aimed to test an aspect of auditory processing that we thought to reflect vulnerability to increased demands of the listening situation. Processing capacity can be taxed in experimental designs by making the tasks themselves more demanding, or adding other factors such as noise to disturb the performance (Magimairaj et al., 2021). Increasing processing demands in various ways has been associated with performance degradation, which affects children with language difficulties proportionally more than their typically developing peers (Coady et al., 2007; Ziegler et al., 2005). Our study design also allowed investigating whether auditory processing reflected by overall ERPs to sine tones or MMN, across manipulations of noise and predictability, would be associated with language abilities in our sample.

#### MMN and ERPs in studying procedural learning

Given the proposed domain general nature of procedural memory, in this study, we attempted to expand the evidence from the motor SRT tasks to evidence from an auditory MMN paradigm that measures a form of procedural learning on a neural level, without a component of motor performance. We utilized the design by Lecaignard et al. (2015). They measured ERPs on neurotypical adults, modifying the predictability of deviant stimuli while keeping the probability of the deviants constant. Stimulus predictability was modulated so that in the unpredictable condition, the deviant stimuli occurred pseudo-randomly whereas in the predictable sequences, the order followed a rule with the number of standard stimuli between deviants increasing progressively (two standards–one deviant, three standards–one deviant, four standards–one deviant, and so on until eight standards preceding a deviant). With this paradigm, Lecaignard et al. observed a (1) decreased MMN during predictable sequences (compared to unpredictable tone sequences), and a similar effect within 70 ms after deviance onset, and also (2) a reduced P3a (a positive brain potential at approximately 250–280 ms after stimulus onset, typically associated with stimulus novelty and attentional orienting) for predictable deviants. Authors interpreted these effects as correlates of higher-level implicit learning of statistical structures in the sequence. As the task measures implicit learning of patterns and regularities, it can be seen as a measure of procedural learning. To this end, we used this task to investigate the relationship between procedural learning and language, specifically examining whether the effect of predictability (i.e., the difference in MMN responses between predictable and unpredictable conditions) is more pronounced in children with stronger school-age language abilities than in those with weaker language abilities. We also investigated the effect of procedural learning on children with a history of LT or TED. Specifically, we aimed to determine whether LT/TED group status is associated with procedural memory or whether the language abilities of children with or without a history of LT are modulated by the effect of procedural learning.

### The Aim of This Study

In this study, we aimed to investigate two different accounts of individual differences in language abilities: procedural learning and auditory processing. We tested the two by manipulating predictability (reflecting procedural learning) and presence of noise (reflecting auditory processing). Based on the PDH, we investigated the effect of predictability on MMN and whether this effect would be associated with (1) the history of LT or (2) school-age language abilities. If LT and school-age language ability show similar association between MMN amplitude, the results suggest that similar mechanisms underlie both late talking and school-age language abilities. Similarly, we examined the extent to which MMN is modified by noise (as a measure of auditory processing) and how this effect would be associated with (1) the history of LT or (2) school-age language abilities, possibly supporting the accounts of auditory processing deficits underlying language disorders.

## MATERIALS AND METHODS

### Participants

#### Recruiting

Participants were recruited from the Southwestern Birth Cohort Study (Lagström et al., 2013) including a total of 9,936 children, 1,827 of whom participated in follow-up studies. Current study was part of this cohort study, and both of these were approved by the ethics committee of the Hospital District of Southwest Finland. Informed consent was obtained from all participants and their parents. The participants in this study were the same as in Kautto et al. (2021) and Kautto and Mainela-Arnold (2022).

To reach participants with varying language abilities, we oversampled children with a history of late talking, based on earlier time points of the cohort study. This study included data from 60 participants, aged 7;5 to 10;5 (yr;mo). Half of the participants (*n* = 30) in this study were identified as late talkers at ages 24–36 months. LT was defined as performance 1.25 *SD* or below age expectations on at least one of the following measures: (a) the MacArthur-Bates Communicative Development Inventory (*n* = 14; Fenson et al., 2007; Finnish version Lyytinen, 2000); (b) the Fox Language Inventory (*n* = 15; Korpilahti & Eilomaa, 2002), or (c) the Renfrew Word Finding Vocabulary Test (*n* = 4). In addition to these, two children with missing information from earlier time points of the study were identified as late talkers based on early speech-language service delivery according to parent reports. In addition to the LT group, we had a group of children with typical early development (TED; *n* = 30). These children were required to have no known history of late talking according to parent report and to exhibit performance between −1 and +1 *SD* from population mean in MacArthur-Bates Communicative Development Inventory at 24 months. One child in the control group had no MacArthur Bates Communicative Development Inventory data available but performed within normal limits in the Fox Language Inventory, the Renfrew Word Finding Vocabulary Test, and Reynell Developmental Language Scales III language comprehension at the age of 36 months.

In total, 60 participants (22 girls, 38 boys) were included in this study. All participants had normal hearing according to pure tone audiometry screening (one participant had the hearing level of 30 dB on one ear at 1000 Hz but otherwise passed the screening and had normal hearing according to parent report), Performance Reasoning Index score above 70 as measured by the Finnish version of the Wechsler Intelligence Scale for Children (WISC-IV; Wechsler, 2003), and no frank emotional, behavioral, motor, intellectual, or neurological disability based on parent reports. More details on inclusion and exclusion criteria and LT definition are provided in Kautto et al. (2021) and Kautto and Mainela-Arnold (2022).

#### Language status at school age

Language status at school age was measured using the Narrative Memory and Comprehension of Instructions subtests from NEPSY-II (Developmental Neuropsychological Assessment; Korkman et al., 2007) and the WISC-IV Vocabulary subtest. Individual Language Index scores were computed as a mean of standard scores on these three subtests. Language Indexes together with demographic information in LT and control groups are presented in Table 1.

**Table 1.** Comparison of participants with and without a history of late talking in demographic measures. *P* values from *t* tests between the groups.

	Typical early development (*N* = 30)	Late talker (*N* = 30)	All participants (*N* = 60)	*p* value
Gender	Girls *n* = 11, Boys *n* = 19	Girls *n* = 11, Boys *n* = 19	Girls *n* = 22, Boys *n* = 38	
Age (yr;mo)				
Mean (*SD*)	8;10 (0;7)	9;2 (0;10)	9;0 (0;9)	0.097
Range	8;1–10;2	7;5–10;5	7;5–10;5	
Language Index				
Mean (*SD*)	10.09 (2.78)	8.38 (2.87)	9.23 (2.93)	0.022
Range	5.33–14.67	3.67–13.67	3.67–14.67	
Number of participants by language status				
Weak (language index <−1 *SD*)	*n* = 4 (13.3%)	*n* = 10 (33.3%)	*n* = 14 (23.3%)	
Average	*n* = 20 (66.7%)	*n* = 17 (56.7%)	*n* = 37 (61.6%)	
Strong (language index >=+1 *SD*)	*n* = 6 (20.0%)	*n* = 3 (10.0%)	*n* = 9 (15.0%)	
Performance Reasoning Index				
Mean (*SD*)	108.77 (18.11)	103.13 (16.71)	105.95 (17.51)	0.215
Range	71–140	71–131	71–140	

### EEG

The participants listened to series of predictable and unpredictable tones in a passive oddball paradigm, in silence and with background noise. EEG was recorded with a Bittium NeurOne system using 500 Hz sampling rate with an EEG cap with 32 channels and mastoid references. Impedances were brought near 5 kohm, or below. During the recording, the participants were seated in an arm chair, listening to auditory stimuli presented via headphones and watching a silent movie of their choice. Participants were encouraged to sit silently and ignore the sounds from the headphones. Each EEG recording session took approximately 1.5 hr, including a short break at the midpoint of the experiment. Clinical evaluation of language and performance reasoning were conducted on a separate visit.

#### Stimuli and procedure

The experimental setting was adapted from Lecaignard et al. (2015). The EEG recording consisted of eight blocks, half of which were predictable and half unpredictable stimulus series. Each series consisted of 500 Hz and 550 Hz sine tones (length 70 ms, including 5 ms onset and offset). Half of the blocks had the lower tone and half the higher tone as the standard stimulus. Each combination of predictability and standard pitch was presented both with and without white noise, resulting in eight different stimulus blocks. The order of the blocks was randomized across participants. Both the predictable and the unpredictable sequences had the same probability (17%) of deviant stimulus, but in the predictable sequences the order of the stimuli followed a pattern with an increasing number of standard stimuli in between the deviants (two standards, one deviant, three standards, one deviant, four standards, one deviant, and so on until eight standards precede a deviant).

#### EEG preprocessing

We used MATLAB version R2021a with EEGLAB (Delorme & Makeig, 2004) in data preprocessing. The first 20 s of each block were removed because we were interested in how participants learn regularities and expected the beginnings of the blocks that introduce new patterns not to reflect learning. Data from all eight experiment blocks from each participant were then combined for preprocessing. Sampling rate was changed to 250 Hz. Channels with poor recording quality were removed using the pop_rejchan function with maximum 4 *SD* threshold limit using probability, kurtosis, and spectrum measures for each channel. A high-pass filter (Hamming windowed sinc FIR) was then applied with a 0.5 Hz lower edge of the frequency pass band. Line noise was removed with the ZapLine tool (de Cheveigné, 2020). Reference was set to average. After cutting to epochs (−200–600 ms), baseline correction was performed with latency range from −200 to 0 ms. Outlier epochs were rejected with an activity probability limit of 4 *SD* for both individual channels and all activities grouped (pop_jointprob function). Independent component analysis (ICA) was performed and components were classified using the ICLabel tool (Pion-Tonachini et al., 2019) after which all components not labeled as brain based with >80% probability were removed. After the ICA, missing electrodes were interpolated using spherical method and reference electrode changed to linked mastoids. The number of interpolated electrodes varied from 0 to 8 (mean 3.78). For the ERP analyses, the blocks with the higher frequency deviant stimuli were combined with the blocks with the lower frequency deviant stimuli. Total number of trials per participant and condition (each combination of standard vs. deviant, predictable vs. unpredictable, and noise vs. no noise) ranged from 84 to 404 (mean 194–200 per condition). As each trial type (standard or deviant) was presented across all other manipulations, total trial counts per participant and condition were high. For example, each participant had data for 415 to 1,188 standard (mean 791.0) and 426 to 1,183 deviant (mean 790.7) stimuli. Detailed information about trial counts is presented in Supplementary Table 1 available in the Supporting Information at https://doi.org/10.1162/nol_a_00129.

### Statistical Analyses

**Figure 1.**
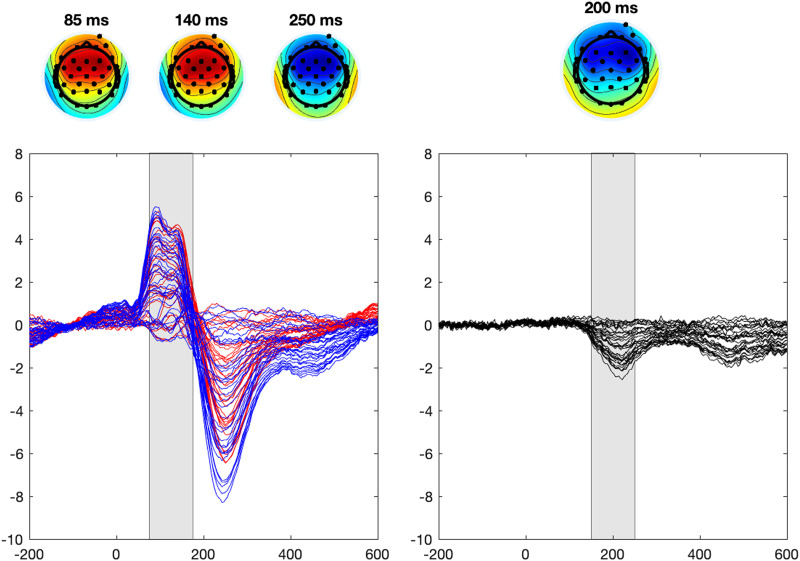
Mean event-related potential (ERP) to sine tone stimuli (left) and difference wave (standard vs. deviant stimuli), with scalp distributions in the peak time points at 85, 140, and 250 ms (ERP, left), and 200 ms (mismatch negativity [MMN], right). Shaded area in MMN figure represents the time window chosen based on the MMN response. Shaded area in ERP waveform represents the post hoc time window (75–175 ms). Responses to standard stimuli presented in red and to deviant stimuli in blue.

Statistical analysis was based on the data from 150–250 ms (the MMN response) time window from the electrode cluster of F3, Fz, F4, FC5, FC1, FC2, and FC6. Because the experimental design was based on the MMN response, the statistical analysis was focused on the time window and electrodes that showed the strongest MMN responses (Figure 1, right). Trials with amplitudes lower than −150 *μ*V and higher than 150 *μ*V were excluded from the analysis as voltages outside this range do not reflect brain-based activity. Only the standards preceding the deviants were taken into account in statistical analyses. Scripts used for data preprocessing are available at https://osf.io/7ng5h/.

The analyses were performed using R software (R Core Team, 2019) with packages lme4 (Bates et al., 2015, p. 4) and lmerTest (Kuznetsova et al., 2017). Packages ggplot2 (Wickham, 2016), sjPlot (Lüdecke, 2019), and ggeffects (Lüdecke, 2018) were used for visualizing the results. Linear mixed effects models of ERP amplitudes on single trial level (i.e., 1,712–2,606 trials per participant) in chosen electrode clusters were modeled as a function of stimulus type (standard vs. deviant), noise, predictability, history of late talking (categorical), and participant language abilities (Language Index calculated as a mean of standardized subtests of different areas of language abilities), and all maximum three-level interactions of these predictors. Language index was centered to population mean but, to preserve the original scale in order to make point estimates easier to interpret, not z-transformed. Participant intercept with slope for stimulus type (standard vs. deviant) was applied as a random factor to account for individual variance in the earlier time window.

## RESULTS

**Figure 2.**
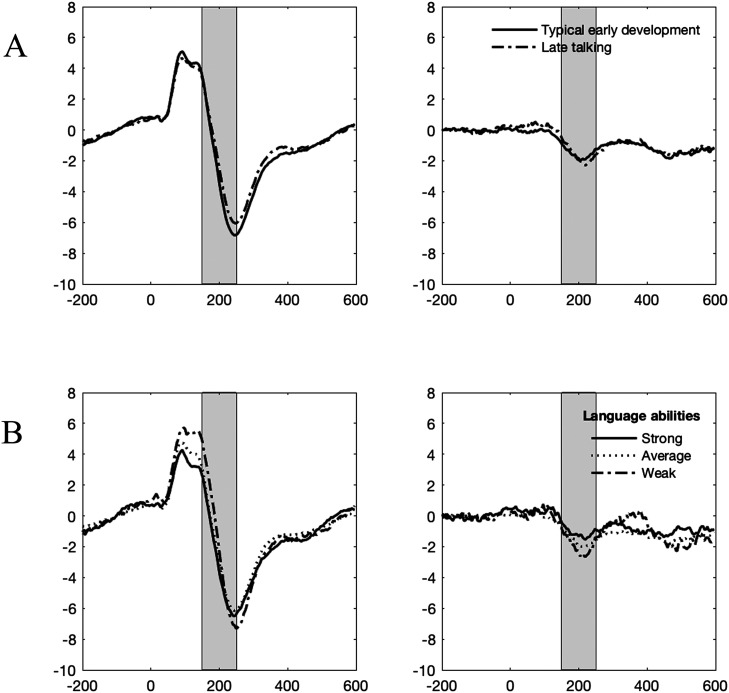
ERP (left) and mismatch response (right) grand average waveforms from the selected electrode cluster visualized by (A) history of late talking and (B) school-age language status. Language abilities are categorized as weak (Language Index <7), average (Language Index 7–13), and strong (Language Index >=13). Gray areas highlight the time window based on MMN response (150–250 ms).

To visualize the differences in the ERP and MMN waveforms in children with varying histories of late talking and language abilities, grand average waveforms from the selected electrode cluster is presented in Figure 2 for early language development (upper row) and school-age language status (lower row). Different stimulus groups are presented in Supplementary Figure 1. Note that participant grouping is done for illustrative purposes only and actual analyses take language abilities into account as a continuous variable, providing more precise estimates of average ERP amplitudes in selected time windows. Summary for the regression model is presented in Table 2.

**Table 2.** Model summary for time window 150–250 ms. Reference levels for categorical variables were standard stimuli, predictable condition, no noise, and typical early development. Language Index was centered to population mean. **Bolded** interaction terms test our research questions.

Predictors	Mean amplitude (*μ*V)			
	Estimate	95% CI	*t* value	*p*
(Intercept)	−2.49	−3.24 – −1.75	−6.55	**<0.001**
Language Index	−0.84	−1.63 – −0.05	−2.09	**0.037**
Stimulus type	−1.56	−2.17 – −0.94	−4.97	**<0.001**
Predictability	−0.39	−0.87 – 0.09	−1.57	0.115
Noise	1.07	0.59 – 1.55	4.33	**<0.001**
Late talking	0.84	−0.29 – 1.97	1.46	0.146
Language Index × Stimulus type	0.41	−0.17 – 0.98	1.40	0.163
Language Index × Predictability	0.05	−0.35 – 0.45	0.24	0.810
Language Index × Noise	−0.11	−0.51 – 0.29	−0.55	0.585
Language Index × Late talking	0.68	−0.40 – 1.75	1.23	0.217
Stimulus type × Predictability	−0.21	−0.84 – 0.42	−0.65	0.515
Stimulus type × Noise	0.42	−0.21 – 1.06	1.30	0.192
Stimulus type × Late talking	−0.98	−1.88 – −0.08	−2.14	**0.032**
Predictability × Noise	0.09	−0.55 – 0.72	0.27	0.788
Predictability × Late talking	0.01	−0.67 – 0.69	0.03	0.980
Noise × Late Talking	−0.75	−1.43 – −0.06	−2.14	**0.032**
**Language Index × Stimulus type × Predictability**	0.26	−0.14 – 0.66	1.28	0.201
**Language Index × Stimulus type × Noise**	0.05	−0.35 – 0.45	0.23	0.817
Language Index × Stimulus type × Late talking	−0.94	−1.65 – −0.24	−2.63	**0.009**
Language Index × Predictability × Noise	0.17	−0.24 – 0.57	0.81	0.419
Language Index × Predictability × Late talking	−0.18	−0.59 – 0.22	−0.90	0.370
Language Index × Noise × Late talking	−0.20	−0.60 – 0.20	−0.97	0.332
Stimulus type × Predictability × Noise	−0.29	−1.02 – 0.45	−0.76	0.447
**Stimulus type × Predictability × Late talking**	0.82	0.04 – 1.59	2.06	**0.040**
**Stimulus type × Noise × Late talking**	0.66	−0.12 – 1.44	1.66	0.096
Predictability × Noise × Late talking	−0.10	−0.87 – 0.68	−0.25	0.806

### Overall Responses and Effects of Noise and Predictability

The estimated ERP amplitude (of a child with TED, with average Language Index) to predictable standard stimulus in silence was −2.49 *μ*V. The statistically significant main effect of stimulus type indicates the presence of MMN: on average, responses to deviant stimuli were −1.56 *μ*V more negative than to standard stimuli. Predictability did not significantly affect overall ERPs or the magnitude of MMN (Stimulus type × Predictability), indicating that unlike Lecaignard et al. (2015), we did not observe the hypothesized ERP correlate of pattern learning (measuring procedural memory). Responses to stimuli in noise were on average 1.07 *μ*V weaker than in silence.

### Effects Related to Language

**Figure 3.**
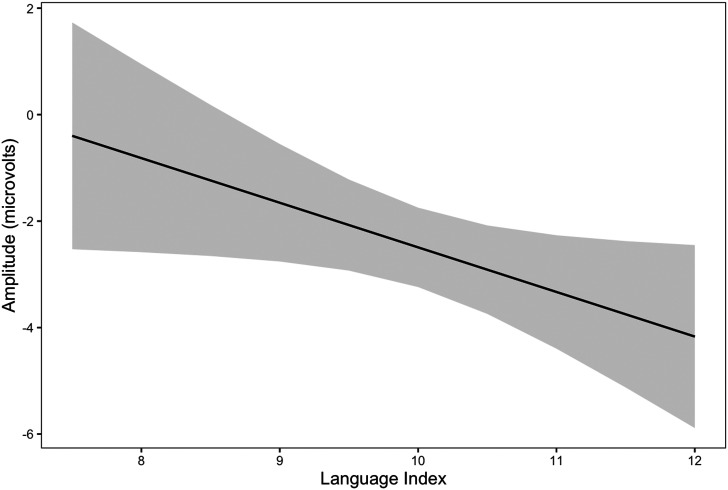
Overall ERP amplitudes were associated with Language Index at 150–250 ms time window. Gray areas represent 95% confidence intervals.

**Figure 4.**
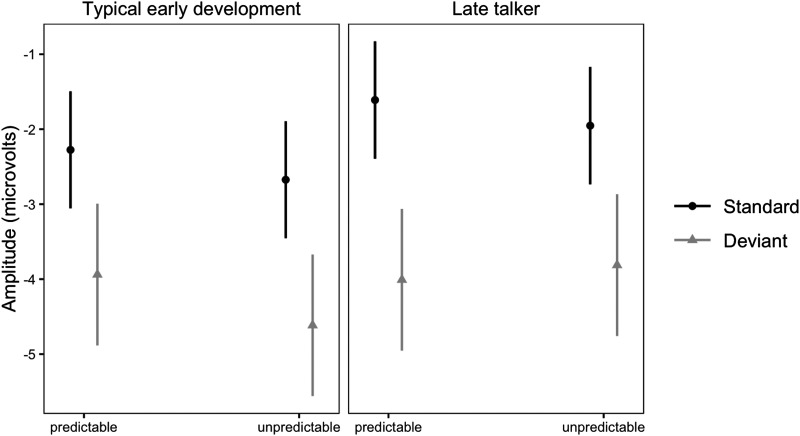
Estimated ERP amplitudes and the difference between standard and deviant stimuli (reflecting MMN) in the 150–250 ms time window as a function of predictability and history of late talking. In children with typical early development, the MMN was larger in unpredictable condition as compared to predictable, but in children with a history of late talking, MMN was larger in predictable than in unpredictable condition. Error bars represent 95% confidence intervals.

**Figure 5.**
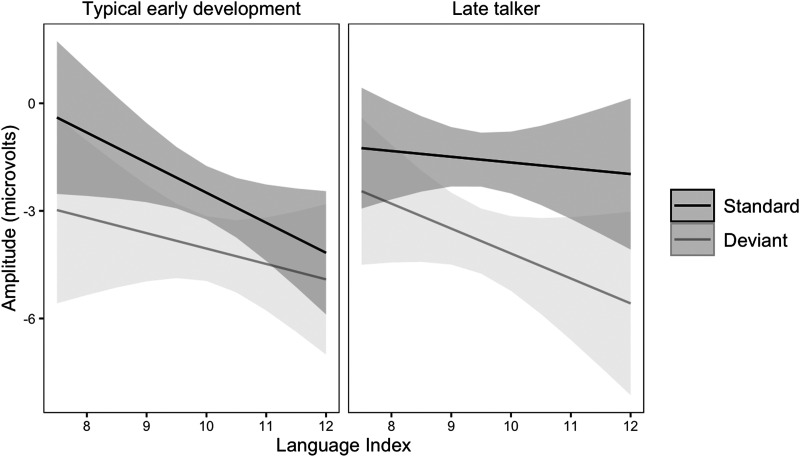
Estimated ERP amplitudes and the difference between standard and deviant stimuli (reflecting MMN) in the 150–250 ms time window as a function of Language Index and history of late talking. In late talkers, the MMN was slightly larger in children with stronger school-age language abilities, but in children with TED direction of the interaction was even the opposite. Gray areas represent 95% confidence intervals.

Language Index was significantly associated with ERP amplitudes: participants with stronger language abilities showed stronger overall responses to stimuli (Figure 3). Contrary to our hypotheses, we did not observe associations between school-age language abilities and effect of noise on MMN (Language Index × Stimulus type × Noise), or effect of predictability (Language Index × Stimulus type × Predictability). Consistent with the hypotheses, an interaction of MMN, predictability and history of late talking was observed. In children with TED, the MMN was larger in the unpredictable condition as compared to the predictable, but in LTs, MMN was larger in the predictable than in the unpredictable condition (Figure 4). The effect of noise to ERPs was larger in children with TED as compared to children with LT (Noise × Late talking). In late talkers, the MMN was slightly larger in children with stronger language abilities whereas in the TED group, similar effect was not observed or the MMN was even smaller on the children with stronger language abilities (Figure 5). However, the effect size was small and in the TED groups the 95% condifence intervals for standard and deviant stimuli had relatively high overlap.

### Post Hoc Analysis in the P1 Time Window

In the MMN time window (150–250 ms), we observed an association between general ERP amplitude and language abilities, rather than MMN and language ability. However, given that the time window was chosen based on the MMN response (not the general, stimulus-evoked response; see Figure 2, left), we also wanted to test if language abilities would associate with early stimulus-evoked activation. We thus modelled the P1 response in the 75 to 175 ms time window from stimulus onset (Figure 1, left). The electrodes and modelling procedure were similar to the statistical model in the MMN-based time window, but the model did not include experimental manipulations (standard vs. deviant, noise or predictability) or late talking as predictors. However, stimulus type (i.e., MMN amplitude) was included in the random effects parts of the model to account for possible individual differences in the responses to this contrast. In addition to language abilities, we added age as a predictor to the model to account for possible effects of age, as the auditory processing in this age group might be related to the neural maturation level (Bishop et al., 2011; Kihara et al., 2010). Age was fitted as a continuous predictor, scaled and centered to sample mean.

**Figure 6.**
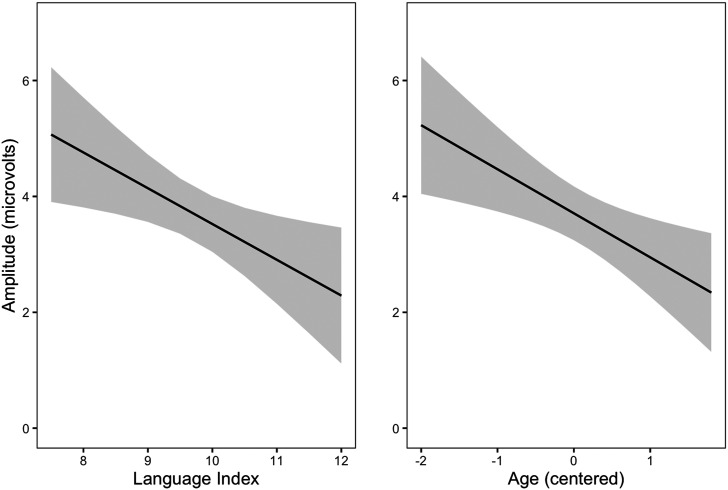
The P1 amplitude in the 75–175 ms time window decreased as a function of language abilities (left) and age (right). Age is scaled to z-values and centered to sample mean, −2 corresponds roughly to 7;4 (yr;month), 0 to 9;0, and 1 to 9;10. Gray areas represent 95% confidence intervals.

Summary for the post hoc model is presented in Table 3. The estimated amplitude for a child with average Language Index and sample average age was 3.55 *μ*V. This ERP amplitude was associated with the main effects of language abilities and age so that children with higher language abilities or age had weaker (more negative) responses (Figure 6). No significant interaction between language abilities and age was observed.

**Table 3.** Post hoc model summary (P1 in time window 75–175 ms). Language Index and age were centered to population and sample mean, respectively.

Predictors	Mean amplitude (*μ*V)			
	Estimate	95% CI	*t* value	*p*
(Intercept)	3.55	3.06–4.03	14.43	**<0.001**
Language Index	−0.63	−1.10 – −0.15	−2.56	**0.010**
Age	−0.69	−1.27 – −0.12	−2.35	**0.019**
Language Index × Age	0.25	−0.29–0.80	0.91	0.362

## DISCUSSION

In this study, we investigated whether experimental manipulations targeting procedural learning and perceptual load differently affect auditory mismatch responses in children with varying language abilities. Based on the PDH, we hypothesized that the effect of predictability on the MMN would be larger in children with stronger as compared to those with weaker language abilities, and that this effect would also differentiate between late talkers with persistent and recessive language difficulties. Based on the auditory processing theories of language development, we hypothesized that noise would decrease MMN to sine tones especially in children with weak language abilities, and that this decrease could also differentiate between former late talkers and children with TED. Our results provided little support for our hypotheses on the effects of predictability or noise. However, in line with auditory processing theories, weaker response at 75–175 ms and stronger response at 150–250 ms to sine tones in general were associated with better school-age language abilities (also visible in the grand average waveforms in Figure 2).

### Procedural Learning and ERPs

The effect of predictability on MMN using a paradigm similar to ours has been earlier observed in an adult sample (Lecaignard et al., 2015), and we expected to observe the same effect in children. However, we did not observe a group-level effect of predictability on MMN. Predictability did not modulate overall ERP responses to sine tones either. We found an effect that differentiated between late talkers and children with TED: in TED children, the MMN was larger in unpredictable than predictable condition—as would have been expected according to our hypotheses based on Lecaignard et al. (2015)—but in children with a history of LT, MMN was larger in predictable than in unpredictable condition (Figure 4). This could be due to LTs showing weaker pattern learning as compared to children with TED. However, this effect was small and, contrary to our hypothesis, not related to school-age language outcomes. In conclusion, we did not find support to procedural deficit hypothesis, measured by the effect of predictability on low-level auditory processing, in predicting LT or school-age language abilities. We were left skeptical about the suitability of the predictability MMN paradigm for testing the hypothesis at least at this point of development, as an expected effect of predictability was only observed in children with LT, and it was not related to school-age language abilities.

### The Effect of Noise on ERPs

In this study, we used noise to modulate auditory processing. In the MMN time window, noise decreased ERP amplitudes in general (compared to silence), but it did not affect the amplitude of the MMN. Overall, the effect of noise on ERPs was observed in children with TED children but not in children with LT. A possible reason for this is that the LT group was heterogenous, including both children with language learning difficulties and children who were late to talk for other reasons. Also, even though the children with and without a history of LT did not significantly differ in age, children in the LT group were slightly older, which could have had a minor effect on ERP and (near significant) MMN differences between the groups.

In contrast to our hypothesis on language abilities and auditory processing measured by the noise manipulation, we did not observe noise to decrease ERPs or MMN responses more in children with weaker as compared to higher school-age language abilities, suggesting no differences between children with varying language abilities in the vulnerability to noise in auditory processing. However, as discussed in the next section, we suggest that overall ERP amplitudes reflect differences in the early stages of auditory processing, also enabling more efficient processing at later stages, between children with strong and weak language abilities.

### ERPs and MMN in Relation to the History of Late Talking and School-Age Language Abilities

We did not observe MMN to be associated with overall language abilities. However, an interaction of LT, language abilities, and MMN (Figure 5) suggested that in the LT group, larger MMN was associated with better school-age language abilities. Overall, the MMN was slightly larger in children with a history of LT. The interaction effect was small and not related to the noise or predictability manipulations. One possible reason is the children with LT being slightly older than the TED children. MMN responses to sine tones have been reported to decrease in latency and be more clearly defined during child development, which might, at least in part, explain our findings of larger MMN in the LT group (Gomot et al., 2000). All in all, LT status did not relate any ERP component in the hypothesized or clearly interpretable way. One possible explanation relates to the length of the time between measurements. Several years had passed between defining the LT status and the ERP measurements. Ideally, toddler ERP measurements would have been conducted together with the school-age ERP measurements.

In the P1 time window (post hoc, 75–175 ms), stronger ERP responses were associated with weaker language abilities and younger age. The main effects of age and school-age language abilities suggested that the relationship between ERPs and age was similar to the relationship between ERPs and language abilities. Weaker positive ERP amplitudes were associated with better language abilities and higher age. Comparable findings have been reported by Bishop and McArthur (2005), who reported that in a subgroup of children with DLD, auditory ERP waveforms to sine tones resembled those of younger, typically developing children.

The findings of weaker ERPs associated with better school-age language abilities and higher age can be interpreted to be related to effects of attention to ERPs. Attention has been found to correlate with ERP amplitudes, with stronger responses observed for attended stimuli compared to unattended ones (Alho et al., 1989; Näätänen et al., 1978; Novak et al., 1990). In relation to this, we suggest that the stronger responses in children with weaker language abilities may reflect slow development in automatization of the early auditory processing. Children with strong language abilities may have efficient and “cost-effective” early processing, reflected by smaller response amplitudes. In previous research, the P1 amplitude to sine tones has also been reported to decrease with age (Bishop et al., 2011), which could, in line with our interpretation of less effort needed to auditory processing, relate to our findings on the maturation of these responses. Presumably, in our sample older children and children with stronger language abilities had a more mature and efficient auditory processing as compared to younger children and children with weaker language abilities. Differences in processing efficiency may explain why younger participants had stronger P1 responses than older participants.

Consistent with our interpretation focusing on attention, previous studies have reported repetition suppression observed in long experiments with many repetitions of certain stimuli as a decrease in neural activity (Nordt et al., 2016). In an MEG study by Helenius et al. (2014), children without DLD showed larger decrease in responses to repeating word stimuli as compared to peers with DLD. Similar findings were reported by Torkildsen et al. (2009), a larger repetition suppression effect to word stimuli in 20-month-old children with larger as compared to smaller expressive vocabularies. In addition to studies using linguistic stimuli, a recent study also reported larger repetition suppression effect to complex nonlinguistic stimuli in infants whose mothers had stronger reading skills as compared to infants whose mothers’ reading skills were weaker (Cantiani et al., 2023). Our result that weaker P1 amplitude was associated with stronger language abilities is in line with these studies, but suggests that the repetition suppression effect might relate to individual differences in language abilities also in early responses elicted by simple, nonlinguistic auditory stimuli.

In contrast to the post hoc P1 time window (75–175 ms), in the MMN based 150–250 ms time window, better language abilities were associated with overall stronger ERP amplitudes to sine tones. ERPs observed during this time window likely reflect processing higher-level information than our earlier time window. Efficient processing of this higher-level information could necessitate successful processing of the physical features during earlier stages. Another possible explanation is related to the chosen time window, which was based on the MMN peak and likely captured the peak of N2 wave in some but not all participants. This explanation is discussed further in the Chosen Time Windows section, below.

Differences in experimental designs, stimuli, and participant groups complicate comparison of our findings to previous literature. Partly in contrast to our results, McArthur et al. (2009) reported “flatter” ERPs in the N1-P2 region on a significant subgroup of 6- to 12-year-old children with language and reading impairments. In our sample, the P1 wave was stronger and the negative response at 150–250 ms weaker in children with weaker as compared to stronger language abilities. However, we did not observe a clear P2 wave and our latter time window was apparently closer to N2 than P2 wave. Bishop et al. (2012) reported reduction of a positive wave at 88–160 ms in children and adolescents with DLD, which was the opposite of what we observed in the 75–175 ms window. The differing findings could probably be due to the differences in ISIs, which in our study were significantly shorter (540 ms) than in Bishop et al. (825 ms) or McArthur et al. (900 or 975 ms). Children with language impairments have been reported to exhibit more negative N100 peak amplitudes to tones than typically developing peers, especially with short ISIs (Weber-Fox et al., 2010). However, different age groups complicate direct comparison of these study findings since younger children tend to show positive and older children negative responses at this time range.

### Chosen Time Windows

As the ERP maturation continues to adolescence and modulates both the amplitudes and latencies of the responses (Kihara et al., 2010), we did not use exactly the same time windows in our analysis that were used in the Lecaignard et al. (2015) study. Choosing exactly the same time windows would have resulted in missing the interesting ERP components in our sample, because in children the timing of the ERP responses is different than in adults (Bishop et al., 2011). We also aimed to choose time windows that would be suitable to study both the effects of capacity load and predictability. Lecaignard et al. (2015) reported a very early effect of predictability within 70 ms from stimulus onset on adults. As the participants in our sample did not show clear ERP responses at this early latency, we did not include such an early time window in our analyses. We focused our analyses in MMN response-based time window as a clear MMN response was observed in our sample. Lecaignard et al. (2015) also observed an effect of predictability to P3a responses. However, we did not observe a P3a component in our group-level data and thus did not include time window based on P3a in our analyses.

As the MMN based time window (150–250 ms) roughly represented the time between P1 and N2 peaks and our main observation was not related to the contrast between standard and deviant stimuli but ERP responses overall, we focused our post hoc analysis in the time window 75–175 ms, capturing the P1 peak in our population. Neither did the MMN time window fully capture the peak of N2 wave, which was observed approximately at 250 ms (Figure 2). We can not rule out the possibility that the ERP amplitude at this time window differs between children with varying language abilities because of different latencies of the peak responses of N2. Bishop et al. (2011) reported smaller P1 responses followed by earlier (yet weaker) N2 responses in 13- to 16-year-old children, compared with 7- to 12-year-olds. It might be that our time window captures the N2 peak maximum in some children, perhaps those with strong language abilities (thus showing more negative responses), but in some children the peak maximum is reached later than at 250 ms. However, possible differences in response latencies were outside the scope of this study.

## CONCLUSIONS AND FUTURE DIRECTIONS

It is noteworthy that electrophysiological responses to even simple sine tones—not only to complex linguistic stimuli—and at relatively early stages (P1 at 75–175 ms from stimulus onset), were connected to language abilities. Our results support the findings that development of auditory processing (e.g., Kihara et al., 2010) continues to school age, and suggest that this development might have different trajectories in children with low and high language abilities. However, one must be cautious in making conclusions about language development based on this study since this was not a follow-up study on the same participants, and the effect of age was compared only between participants. Auditory processing accounts on language difficulties have gained some support (e.g., Bishop & McArthur, 2005; Corriveau et al., 2007), but a straightforward relationship between the two has also been questioned (e.g., Bishop et al., 1999). Our findings indicate that auditory ERPs to simple sine tones at relatively early stages of processing are associated with language abilities. Weak low-level auditory processing results in difficulty encoding physical features of phonemes. This leads to degraded phonological representations, which in turn snow-ball into difficulties with learning words and grammar, as well as linking meaning with the degraded representations. To gain a deeper understanding of the maturation of auditory ERPs and its relation to language acquisition, we suggest future studies with longitudinal designs to model trajectories of auditory development on children with varying language abilities.

## ACKNOWLEDGMENTS

This research was financially supported by an anonymous endowed fund to the University of Turku Speech-Language Pathology. We thank the Steps to the Healthy Development and Well-being of Children Cohort Study for assistance in recruiting children; Eira Jansson-Verkasalo for her contribution on planning the experiment; Pirjo Korpilahti for helpful discussions; and Teemu Laine, Jessica Åkermarck, and University of Turku Speech-Language Pathology for their assistance in data collection. Finally, we thank the children and families who participated.

## FUNDING INFORMATION

Anna Kautto, University of Turku Graduate School (https://dx.doi.org/10.13039/501100019391).

## AUTHOR CONTRIBUTIONS

**Anna Kautto**: Conceptualization: Supporting; Data curation: Equal; Formal analysis: Lead; Methodology: Equal; Project administration: Equal; Writing – original draft: Lead. **Henry Railo**: Formal analysis: Equal; Methodology: Equal; Supervision: Equal; Writing – review & editing: Lead. **Elina Mainela-Arnold**: Conceptualization: Lead; Project administration: Lead; Supervision: Equal; Writing – review & editing: Equal.

## DATA AVAILABILITY STATEMENT

The code used for data preprocessing and statistical models is available at https://osf.io/7ng5h/. Due to ethical issues and institutional restrictions, the data are only available on request from the authors.

## Supplementary Material


