# Supplementary material for: Low-Level Auditory Processing Correlates With Language Abilities: An ERP Study Investigating Sequence Learning and Auditory Processing in School-Aged Children
Source: Neurobiol Lang (Camb). 2024 Jun 3;5(2):341–59. doi: 10.1162/nol_a_00129 (PMC11093401; doi:10.1162/nol_a_00129)
Supplement: Supplementary file 1 [file nol-5-2-341-s001.pdf]

SUPPLEMENTARY MATERIAL

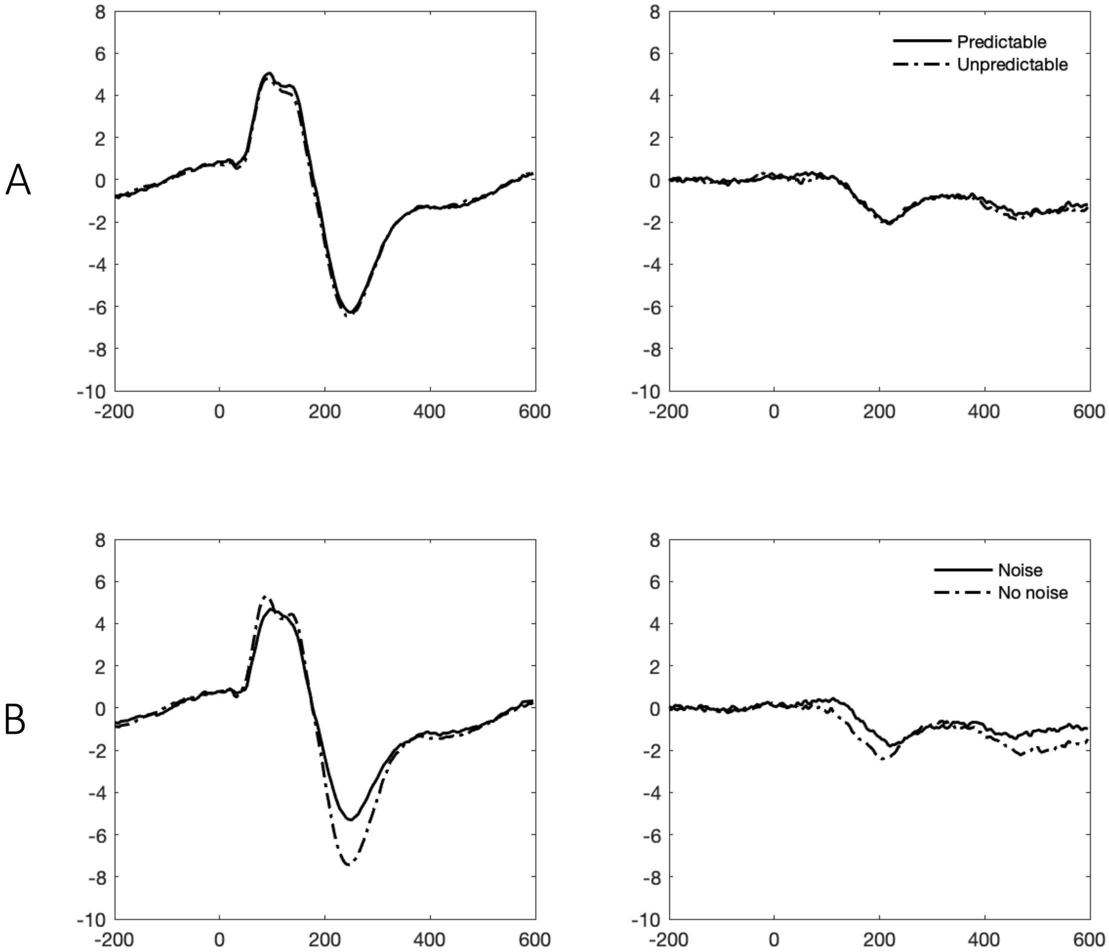

Supplementary Figure 1: ERP (*left) and MMN* (*right) grand average waveforms from the selected electrode cluster visualized for stimuli in predictable and unpredictable sequences* (*A) and with or without noise* (*B).*

Supplementary Table 1: Trial counts per participant and condition for statistical models.

Stimulus type	Predictability	Noise	Minimum	Maximum	Mean
standard	predictable	no	84	306	198
standard	predictable	yes	98	314	194
standard	unpredictable	no	94	403	200
standard	unpredictable	yes	99	390	199
deviant	predictable	no	84	298	197
deviant	predictable	yes	95	315	194
deviant	unpredictable	no	96	404	200
deviant	unpredictable	yes	111	389	199
